# Trends of machine learning for dental caries research in Southeast Asia: insights from a bibliometric analysis

**DOI:** 10.12688/f1000research.154704.2

**Published:** 2024-10-03

**Authors:** Faizul Hasan, Hendrik Setia Budi, Lia Taurussia Yuliana, Mokh Sujarwadi

**Affiliations:** 1 Faculty of Nursing, Chulalongkorn University, Bangkok, Bangkok, 10330, Thailand; 2 Department of Oral Biology, Dental Pharmacology, Faculty of Dental Medicine, Universitas Airlangga, Surabaya, East Java, 60132, Indonesia; 3 Faculty of Nursing, Universitas Jember, Jember, East Java, 68121, Indonesia

**Keywords:** dental caries; transmissible disease; health and well-being; machine learning; artificial intelligence

## Abstract

**Background:**

Dental caries is a common chronic oral disease, posing a serious public health issue. By analyzing large datasets, machine learning shows potential in addressing this problem. This study employs bibliometric analysis to explore emerging topics, collaborations, key authors, and research trends in Southeast Asia related to the application of machine learning in dental caries management.

**Methods:**

A comprehensive selection using the Scopus database to obtain relevant research, covering publications from inception to July 2024 was done. We employed the Bibliometric approaches, including co-authorship networks, yearly publishing trends, institutional and national partnerships, keyword co-occurrence analysis, and citation analysis, for the collected data. To explore the visualization and network analysis, we employed the tools such as VOSviewer and Bibliometrix in R package.

**Results:**

The final bibliometric analysis included 246 papers. We found that Malaysia became the top contributor with 59 publications, followed by Indonesia (37) and Thailand (29). Malaysia had the highest Multiple Country Publications (MCP) ratio at 0.407. Top institutions including the Universiti Sains Malaysia led with 39 articles, followed by Chiang Mai University (36) and the National University of Singapore (30) became the leader. Co-authorship analysis using VOSviewer revealed six distinct clusters. A total of 1220 scholars contributed to these publications. The top 10 keywords, including ‘human’ and ‘dental caries,’ indicated research hotspots.

**Conclusion:**

We found growing evidence of machine learning applications to address dental caries in Southeast Asia. The bibliometric analysis highlights key authors, collaborative networks, and emerging topics, revealing research trends since 2014. This study underscores the importance of bibliometric analysis in tackling this public health issue.

## Introduction

Dental caries (DC), a common chronic oral disease, is a substantial threat to global health, with high incidence rates despite disease prevention efforts ( Cheng et al., 2022). individuals encountering food insecurity are significantly more prone to dental caries, as evidenced by meta-analyses that demonstrate a higher risk of caries in food-insecure individuals compared to those with food security ( Drumond, De Arruda, Bernabé, Mesquita, & Abreu, 2023). Both clinical researchers and dental practitioners are rigorously investigating optimal therapeutic interventions for this condition.

DC continue to be a major oral health concern in the Southeast Asia Region (SEAR), impacting children aged 5 to 15 ( Kale, Kakodkar, Shetiya, & Rizwan, 2019). Studies have demonstrated an elevated prevalence of DC among children in SEAR countries, with variability among age groups and nations ( Kale et al., 2019). In these investigations, the dentition status based on the 1997 WHO criteria was the most widely employed index for evaluating DC ( Kale et al., 2019). Furthermore, the burden of DC extends to Cambodian preschool children, with a significant proportion developing carious lesions and pulpally affected teeth, underlining the critical need for intervention to address Early Childhood Caries (ECC) in the region ( Turton, Chher, Sabbah, Durward, Hak, & Lailou, 2019). Efforts to integrate oral health into national health systems and establish uniform treatment standards are critical steps toward combating the prevalence of DC in the SEAR area ( Acharya, Mathur, Tadakamadla, & Brand, 2024).

To increase diagnostic accuracy and prevent caries from being neglected, computer-based intelligent vision systems employing deep learning techniques have been developed, with a remarkable accuracy rate of 99.13% in automatic caries diagnosis based on periapical images ( Imak, Celebi, Siddique, Turkoglu, Sengur, & Salam, 2022). In addition, innovations in caries detection include self-training-based approaches exploiting small sets of labeled images and enormous quantities of unlabeled images, boosting caries detection and segmentation performance without the requirement for extensive annotated data ( Qayyum et al., 2023).

The current state of research in Southeast Asia regarding the application of machine learning techniques to DC research is lacking in comprehensive studies that incorporate multimodal data, including behavioral factors, radiographic imaging, and microbial analysis, to improve treatment outcomes and caries risk assessment ( Jusman, Anam, Puspita, & Saleh, 2021; Velmurugadass, Rohith, Harish, & Reddy, 2023; Wu et al., 2022). While machine learning models such as Support Vector Machine (SVM) and K-Nearest Neighbors (KNN) have been effectively utilized in dental caries classification research, there remains a dearth of understanding regarding the distinct challenges and determinants that impact DC within Southeast Asian communities, notably in nations such as Thailand and Indonesia ( Ula, Anjani, Ulva, Sahputra, & Pratama, 2022; Wu et al., 2022). Researchers can better understand the complex etiology of DC in this region through the application machine learning techniques to a wide range of data sources, including genetic, behavioral, and environmental factors.

Bibliometric analysis connects research and practice, ensuring researchers are informed by pertinent and influential literature, while clinicians are equipped to execute evidence-based interventions efficiently. By conducting a thorough assessment of literature utilizing machine learning methodologies, we have provided a comprehensive overview of the themes, patterns, and bibliometric attributes that define this body of literature from 2002 to 2024. The outcomes derived from our research unveil significant findings and propose innovative pathways for further exploration within this rapidly expanding realm of knowledge acquisition and academic inquiry.

## Methods

### Search strategy

We conducted a search for pertinent articles within the Scopus database, recognized as one of the most extensive international repositories of scholarly publications. Scopus encompasses an estimated 24,000 present-day periodicals sourced from over 5,000 global publishers, encompassing peer-reviewed journals, conference papers, periodicals for commercial purposes, and scholarly book series. The database comprises roughly 75 million entries, spanning various forms of scholarly outputs such as journals, conference proceedings, patents, and publications covering a wide array of disciplines. Our search for relevant literature involved the utilization of specific keywords: (ALL ((“artificial intelligence” OR “machine intelligence” OR “robot*” OR “robot technology” OR “assistant robot” OR “robot-assisted" OR “computational intelligence” OR “computer reasoning” OR “deep learning” OR “computer vision system” OR “sentiment analysis” OR “machine learning” OR “neural network*” OR “data learning” OR “expert* system*” OR “natural language processing” OR “support vector machine*” OR “decision tree*” OR “data mining” OR “deep learning” OR “neural network*” OR “bayesian network*” OR “intelligent learning” OR “feature* learning” OR “feature* extraction” OR “feature* mining” OR “feature* selection” OR “unsupervised clustering” OR “image* segmentation” OR “supervised learning” OR “semantic segmentation” OR “deep network*” OR “neural learning” OR “neural nets model” OR “graph mining” OR “data clustering” OR “big data” OR “knowledge graph”)) AND ALL ("Dental caries” OR “Caries, Dental” OR “Dental Cavity” OR “Dental Decay” OR “Tooth Decay” OR “Decay, Tooth” OR “Tooth Demineralization” OR “Caries, Tooth” OR “Caries, Teeth” OR “Teeth Caries”)) AND PUBYEAR > 2001 AND PUBYEAR < 2025 AND (LIMIT-TO (DOCTYPE, “ar”) OR LIMIT-TO (DOCTYPE, “re")) AND (LIMIT-TO (AFFILCOUNTRY, “Malaysia”) OR LIMIT-TO (AFFILCOUNTRY, “Indonesia”) OR LIMIT-TO (AFFILCOUNTRY, “Thailand”) OR LIMIT-TO (AFFILCOUNTRY, “Singapore”) OR LIMIT-TO (AFFILCOUNTRY, “Brunei Darussalam”) OR LIMIT-TO (AFFILCOUNTRY, “Philippines”) OR LIMIT-TO (AFFILCOUNTRY, “Viet Nam”) OR LIMIT-TO (AFFILCOUNTRY, “Myanmar”) OR LIMIT-TO (AFFILCOUNTRY, “Cambodia”)). Two professionals with a minimum of five years of expertise in conducting systematic reviews endorsed our search strategy subsequent to a meticulous examination of all article titles obtained. They identified all pertinent publications, with any discrepancies being settled through deliberation with an additional author. Ultimately, all articles retrieved were preserved. In addition, we selected data from 2002 to 2024. This timeframe was selected because of technological advancements commencing in 2002, signifying a crucial transformation in research methodologies and data accessibility, enabling the identification of pertinent trends in the domain. Incorporating data through 2024 guarantees the inclusion of the most recent advancements and offers a thorough examination of emerging research. This timeframe offers dependable and accessible data, crucial for a comprehensive bibliometric investigation.

### Screening

Only articles that utilized Machine Learning in their findings and focused on DC were considered for the bibliometric analysis. There were no restrictions based on language. Excluded from the analysis were letters, editorials, conference abstracts, and book chapters, while research papers and reviews published in peer-reviewed journals were included. The evaluation of the articles was conducted by two experts in bibliometric analysis, who possessed extensive experience and assessed the articles according to predetermined criteria and guidelines ( Martinez, Al-Hussein, & Ahmad, 2019).

### Bibliometric analysis

In order to produce replicable and measurable data that is pertinent to policy administration, bibliometric analysis ( Pritchard, 1969) serves as a quantitative assessment of scientific research, examining current research patterns within a specific field ( Mooghali, Alijani, Karami, & Khasseh, 2011). Bibliometric analysis has the capability to offer a comprehensive examination of a specific subject of investigation, along with pinpointing research domains that scholars ought to delve into and methodologies that authors have formulated to achieve their objectives ( Su & Lee, 2010). A holistic representation of the field allows readers to better comprehend patterns and trends in DC research.

Additionally, the distribution, proportions, and frequency for each journal were presented. The categories, proportions, and occurrence for each periodical were presented. The pertinent author ratios, occurrence, and proportion were computed for each country for both individual and multiple nations. The occurrence and citation rate for each author and organization were also provided. The research impact of each nation, periodical, organization, and author was arranged based on the quantity of publications. All information was formulated and depicted utilizing VOSviewer (Leiden University) and Bibliometrix (an R package).

## Results

### Publication output

**Figure 1.**
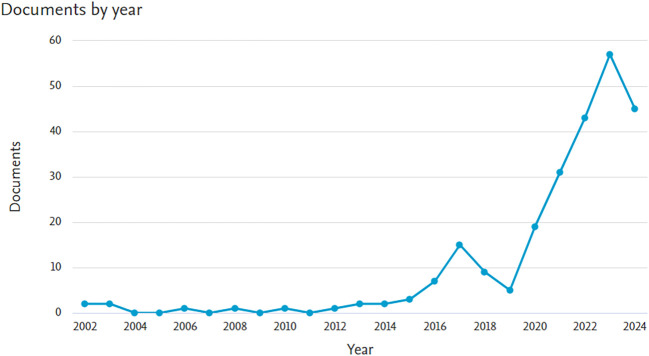
The distribution of articles by year.

The final bibliometric investigation consisted of 246 papers after the screening process. The growth in the quantity of DC articles is visualized in Figure 1.

Table 1 demonstrates the geographical distribution of the leading ten countries that have conducted research on DC. Malaysia emerged as the top contributor with 59 publications, succeeded by Indonesia with 37 publications and Thailand with 29 publications. The country with the highest ratio of Multiple Country Publications (MCP) was Malaysia at 0.407, followed by Singapore at 0.375 and Indonesia at 0.27. Supplementary Figure 1 (Extended data) showcases the network of countries that have collaborated on at least one research project.

**Table 1.** Top 10 countries that published in 2002–2024.

Rank	Country	Articles	SCP	MCP	Freq	MCP_Ratio
1	Malaysia	59	35	24	0.243	0.407
2	Indonesia	37	27	10	0.152	0.27
3	Thailand	29	24	5	0.119	0.172
4	China	17	0	17	0.07	1
5	Singapore	16	10	6	0.066	0.375
6	USA	7	0	7	0.029	1
7	Iran	6	0	6	0.025	1
8	United Kingdom	6	0	6	0.025	1
9	India	5	0	5	0.021	1
10	Japan	5	0	5	0.021	1

MCP = multiple country publications; SCP = single country publications.

Supplementary Table 1 (Extended data) showcases the leading ten institutions that have shown high productivity in conducting research on DC. Among these institutions, Universiti Sains Malaysia stands out as the most prolific university with thirty-nine published articles, followed by Chiang Mai University with thirty-six, and the National University of Singapore with thirty publications.

The utilization of the VOSviewer tool (Leiden University) was employed for conducting co-authorship analysis, aiming to present a thorough depiction of the entire network of countries involved in DC research. The level of collaboration between two countries is determined by the quantity of publications they have co-authored. Out of the total of 8 authors, only 3 authors are interconnected, with each of them having published a minimum of 5 articles. The examination of authorship by country resulted in the identification of six distinct clusters, as illustrated in Supplementary Figure 2 (Extended data).

In the discourse concerning research on DC, a total of 1220 scholars collectively contributed to the publication of 246 scholarly articles. Within the realm of DC research, Table 2 provides a comprehensive overview of the top 10 most prolific authors in this field. Noteworthy among these authors is Li Y, who authored a total of eight scholarly papers on the subject matter.

**Table 2.** Top 10 authors that published in 2002–2024.

Rank	Authors	Articles
1	Li Y	8
2	Gupta R	6
3	Liu Y	6
4	Wantanajittikul K	6
5	Abu PAR	5
6	Chen CA	5
7	Chen SL	5
8	Chen TY	5
9	Fürst T	5
10	Ngoc VTN	5

### Co-occurrence analysis of top 10 keywords

**Figure 2.**
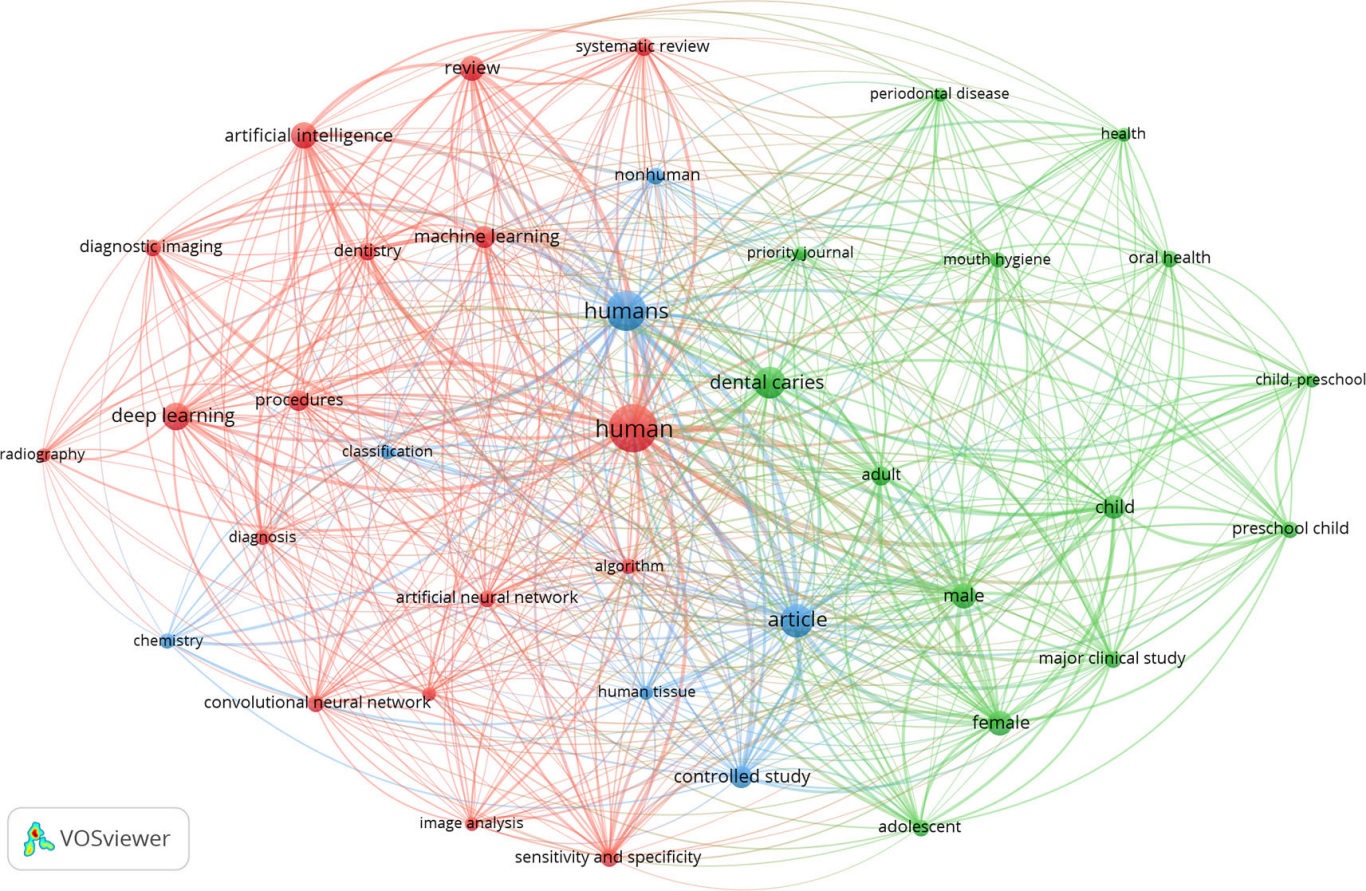
The co-occurrence network of the top 10 keywords in this research, 2002–2024.

The 246 articles were divided into three keyword clusters. The top ten keywords utilized in the articles that were retrieved can be found in Table 3. It is worth mentioning that human, dental caries, article, child, and artificial intelligence were some of the predominant categories. The examination of the co-occurrence of the top 10 phrases revealed the research hotspots in the field of dental caries ( Figure 2).

**Table 3.** Top 10 author keywords 2002–2024.

Rank	Keywords	Occurrences
1	Human	114
2	Humans	79
3	Dental caries	61
4	Article	54
5	Child	52
6	Female	48
7	Male	44
8	Artificial intelligence	41
9	Review	30
10	Adult	28

### Reference co-citation analysis

We delved deeper into the knowledge repository of the DC research domain. Displayed in Table 4 are the top ten articles with high citation counts. Vos T et al., in 2017, garnered the highest number of citations by June 1, 2024, totaling 5476. This particular article was published in LANCET in 2016.

**Table 4.** Top 10 high cited articles 2002–2024.

Rank	First author/Journal	DOI	Total Citations	TC per Year
1	Vos T, 2016, Lancet	10.1016/S0140-6736(16)31678-6	5476	608.44
2	Vos T, 2017, Lancet	10.1016/S0140-6736(17)32154-2	5408	676.00
3	Hay Si, 2017, Lancet	10.1016/S0140-6736(17)32130-X	1590	198.75
4	Shehab M, 2022, Comput Biol Med	10.1016/j.compbiomed.2022.105458	179	59.67
5	Thompson Bc, 2015, Adv Mater	10.1002/adma.201500411	133	13.30
6	Bapat Ra, 2019, Drug Discov Today	10.1016/j.drudis.2018.08.012	101	16.83
7	Hafshejani Tm, 2017, J Control Release	10.1016/j.jconrel.2017.07.041	93	11.63
8	Son Lh, 2018, Biomed Signal Process Control	10.1016/j.bspc.2017.07.005	88	12.57
9	Meglinski Iv, 2010, Laser Phys Lett	10.1002/lapl.200910141	82	5.47
10	Teh Sk, 2008, J Biomed Opt	10.1117/1.2939406	82	4.82

DOI = digital object identifiers; TC = total citation.

## Discussion

To the best of our knowledge, this research is the first bibliometric study to use machine learning to investigate DC. SEAR researchers are becoming more interested in using machine learning technologies to combat DC, according to this bibliometric analysis. The number of papers has increased over the previous ten years (>2014), which highlights the significance of this issue for public health as well as the potential of algorithms that use machine learning to provide assistance.

Machine learning has emerged as a powerful tool in combating dental caries, with Malaysia, Indonesia, and Thailand being at the forefront of utilizing this technology ( Basri, Yazid, Zain, Yusof, Rani, & Zoolfakar, 2024; Wu et al., 2021, 2022). Research conducted in these countries has shown promising results in early caries detection and risk prediction through the application of machine learning algorithms such as artificial neural networks (ANN), convolutional neural networks (CNN), and statistical models ( Basri et al., 2024; Sadegh-Zadeh, Rahmani Qeranqayeh, Benkhalifa, Dyke, Taylor, & Bagheri, 2022; Toledo Reyes et al., 2023; Wu et al., 2022). By integrating multimodal data, including genetic, microbial, demographic, and behavioral factors, these countries have been able to identify key predictors of caries development in both primary and permanent teeth, paving the way for more effective preventive measures and personalized treatment strategies ( Sadegh-Zadeh et al., 2022; Toledo Reyes et al., 2023; Wu et al., 2021, 2022). The collaborative efforts between researchers and healthcare professionals in Malaysia, Indonesia, and Thailand highlight the potential of machine learning in revolutionizing oral health care and addressing the global burden of dental caries.

The research landscape on dental caries utilizing machine learning reveals a notable productivity ranking among institutions. Universiti Sains Malaysia emerges as the most prolific university in this field, boasting thirty-nine published articles, showcasing a strong commitment to advancing knowledge in dental caries detection. Following closely behind is Chiang Mai University with thirty-six publications, indicating a significant contribution to the research domain of dental caries and machine learning. Additionally, the National University of Singapore has also demonstrated substantial engagement in this area with thirty publications, further enriching the scholarly discourse on the early detection and management of dental caries through innovative technological approaches. These institutions’ dedication to research in dental caries using machine learning underscores the global effort to enhance diagnostic accuracy and treatment outcomes in oral healthcare.

In line with previous studies, the co-occurrence of the top 10 phrases in dental caries research, as highlighted in the bibliometric studies ( Melo, Sanz, Forner, Rodríguez-Lozano, & Guerrero-Gironés, 2022; Patil et al., 2020), reveals a strong relationship with the predominant categories of human, dental caries, article, and child. Terms related to the validity of diagnostic methods, tools, and principles used in caries diagnosis, as well as general aspects of caries detection and diagnosis, are commonly found among the top phrases. This suggests that research in dental caries is heavily centered around human subjects, particularly children, with a significant emphasis on diagnostic methods and tools, as well as the overall understanding of the disease. However, interestingly, we found that the use of the keyword ‘artificial intelligence’ is on the rise.

The bibliometric analysis highlights significant research trends, pivotal studies, and deficiencies in the existing literature, which can guide evidence-based public health interventions. By identifying the most commonly cited therapies or successful public health initiatives in dental caries prevention, researchers and policymakers can prioritize these techniques for widespread implementation. The machine learning methodology facilitates the discovery of emerging research domains, enabling the prompt recognition of novel therapies or preventive strategies. This can inform funding distribution and policy formulation, ensuring resources are allocated to the most effective strategies for enhancing oral health outcomes.

Despite the fact that this bibliometric analysis provides interesting information, there are considerable limitations to take into account. The dependency on the Scopus database may result in the omission of significant articles from various sources. The correctness of the metadata associated with the articles also limits the study. Additionally, bibliometric analysis focuses on quantitative rather than qualitative evaluations of research findings and methodologies. Despite these limitations, the results demonstrate the increasing importance of employing machine learning to manage dental caries in Southeast Asia, which may guide future treatments and research areas.

## Conclusion

We found the increasing evidence on the application of machine learning to mitigate the issue of dental caries in Southeast Asia. The bibliometric analysis identifies key authors, collaborative networks, and emerging topics, including dental caries and artificial intelligence. It also reveals growing research trends since 2014. This work underscores the significance of bibliometric analysis in tackling this critical public health challenge.

## Data Availability

No data are associated with this article. Supplementary materials are available in the supplementary data. Supplementary data: https://doi.org/10.5281/zenodo.13138787 ( Faizul, 2024). This project contains the following extended data:
•
Supplementary Fig 1 country 24713.docx
•
Supplementary Fig 2 author 24713.docx
•
Supplementary Table 1 top 10 institution 24713.docx Data are available under the terms of the Creative Commons Zero “No rights reserved” data waiver (CC0 1.0 Public domain dedication). Reporting guidelines: https://doi.org/10.5281/zenodo.13138787 ( Faizul, 2024). STROBE_checklist_v4_combined.docx Data are available under the terms of the Creative Commons Zero “No rights reserved” data waiver (CC0 1.0 Public domain dedication). Software: VOSviewer is a free software tool for constructing and visualizing bibliometric networks. To learn more, visit the VosViewer Getting Started page.

## References

[ref1] Acharya S Mathur MR Tadakamadla SK : Assessing the status of oral health integration in South East Asian Regional Office countries’ Universal Health Coverage–A scoping review. *Int. J. Health Plann. Manag.* 2024;39(2):262–277. 10.1002/hpm.3751 38169038

[ref2] Basri KN Yazid F Zain MNM : Artificial neural network and convolutional neural network for prediction of dental caries. *Spectrochim. Acta A Mol. Biomol. Spectrosc.* 2024;312:124063. 10.1016/j.saa.2024.124063 38394882

[ref3] Cheng L Zhang L Yue L : Expert consensus on dental caries management. *Int. J. Oral Sci.* 2022;14(1):17. 10.1038/s41368-022-00167-3 35361749 PMC8971510

[ref4] Drumond VZ De Arruda JAA Bernabé E : Burden of dental caries in individuals experiencing food insecurity: a systematic review and meta-analysis. *Nutr. Rev.* 2023;81(12):1525–1555. 10.1093/nutrit/nuad031 37040617

[ref22] Faizul H : Dental caries in Asia using ML. Zenodo.[Dataset].2024. 10.5281/zenodo.13138787

[ref5] Imak A Celebi A Siddique K : Dental caries detection using score-based multi-input deep convolutional neural network. *IEEE Access.* 2022;10:18320–18329. 10.1109/ACCESS.2022.3150358

[ref6] Jusman Y Anam MK Puspita S : Machine learnings of dental caries images based on Hu moment invariants features. *Paper presented at the 2021 International Seminar on Application for Technology of Information and Communication (iSemantic).* 2021.

[ref7] Kale SS Kakodkar P Shetiya SH : Dental caries prevalence among 5-to 15-year-old children from SEAR countries of WHO: A systematic review and meta-analysis. *Indian J. Dent. Res.* 2019;30(6):937–947. 10.4103/ijdr.IJDR_654_17 31939375

[ref8] Martinez P Al-Hussein M Ahmad R : A scientometric analysis and critical review of computer vision applications for construction. *Autom. Constr.* 2019;107:102947. 10.1016/j.autcon.2019.102947

[ref9] Melo M Sanz JL Forner L : Current status and trends in research on caries diagnosis: A bibliometric analysis. *Int. J. Environ. Res. Public Health.* 2022;19(9):5011. 10.3390/ijerph19095011 35564406 PMC9102117

[ref10] Mooghali A Alijani R Karami N : Scientometric analysis of the scientometric literature. *Int. J. Inf. Sci. Manag.* 2011;9(1):19–31.

[ref11] Patil SS Sarode SC Sarode GS : A bibliometric analysis of the 100 most cited articles on early childhood caries. *Int. J. Paediatr. Dent.* 2020;30(5):527–535. 10.1111/ipd.12641 32223037

[ref12] Pritchard A : Statistical bibliography or bibliometrics. *J. Doc.* 1969;25:348.

[ref13] Qayyum A Tahir A Butt MA : Dental caries detection using a semi-supervised learning approach. *Sci. Rep.* 2023;13(1):749. 10.1038/s41598-023-27808-9 36639724 PMC9839770

[ref14] Sadegh-Zadeh S-A Rahmani Qeranqayeh A Benkhalifa E : Dental caries risk assessment in children 5 years old and under via machine learning. *Dent. J.* 2022;10(9):164. 10.3390/dj10090164 36135159 PMC9497737

[ref15] Su H-N Lee P-C : Mapping knowledge structure by keyword co-occurrence: A first look at journal papers in Technology Foresight. *Scientometrics.* 2010;85(1):65–79. 10.1007/s11192-010-0259-8

[ref16] Toledo Reyes L Knorst JK Ortiz F : Early childhood predictors for dental caries: a machine learning approach. *J. Dent. Res.* 2023;102(9):999–1006. 10.1177/00220345231170535 37246832

[ref17] Turton B Chher T Sabbah W : Epidemiological survey of early childhood caries in Cambodia. *BMC Oral Health.* 2019;19:1–7. 10.1186/s12903-019-0800-y 31196058 PMC6567398

[ref18] Ula M Anjani FTT Ulva AF : Application of Machine Learning With The Binary Decision Tree Model In Determining The Classification of Dental Disease. *Journal of Informatics and Telecommunication Engineering.* 2022;6(1):170–179. 10.31289/jite.v6i1.7341

[ref19] Velmurugadass P Rohith A Harish M : Dental caries detection system Using R-CNN Algorithm. *Paper presented at the 2023 4th International Conference on Intelligent Engineering and Management (ICIEM).* 2023.

[ref20] Wu TT Xiao J Manning S : Multimodal data integration reveals mode of delivery and snack consumption outrank salivary microbiome in association with caries outcome in Thai children. *Front. Cell. Infect. Microbiol.* 2022;12:881899. 10.3389/fcimb.2022.881899 35677657 PMC9168266

[ref21] Wu TT Xiao J Sohn MB : Machine learning approach identified multi-platform factors for caries prediction in child-mother dyads. *Front. Cell. Infect. Microbiol.* 2021;11:727630. 10.3389/fcimb.2021.727630 34490147 PMC8417465

