# Supplementary material for: Trends of machine learning for dental caries research in Southeast Asia: insights from a bibliometric analysis
Source: F1000Res. 2024 Oct 11;13:908. Originally published 2024 Aug 8. [Version 3] doi: 10.12688/f1000research.154704.3 (PMC11489836; doi:10.12688/f1000research.154704.3)
Supplement: Peer review history

# Reviewer report: Reviewer response for version 3

**DOI:** 10.5256/f1000research.173111.r330860

**Published:** 2024-10-18

**Authors:** Heni Susilowati

**Affiliations:** 1 Department if Oral Biology, Faculty of Dentistry, Universitas Gadjah Mada, Universitas Gadjah Mada, KABUPATEN SLEMAN, DI YOGYAKARTA, Indonesia

After reviewing the revisions made to the manuscript, including major and minor points, I conclude that this article has become more comprehensive. Therefore, I recommend it for final indexing.

 I would like to thank the authors for addressing the suggestions provided in this review.

Is the work clearly and accurately presented and does it cite the current literature?

Yes

If applicable, is the statistical analysis and its interpretation appropriate?

Not applicable

Are all the source data underlying the results available to ensure full reproducibility?

Yes

Is the study design appropriate and is the work technically sound?

Yes

Are the conclusions drawn adequately supported by the results?

Yes

Are sufficient details of methods and analysis provided to allow replication by others?

Yes

Reviewer Expertise:

Oral microbiology and immunology

I confirm that I have read this submission and believe that I have an appropriate level of expertise to confirm that it is of an acceptable scientific standard.

# Reviewer report: Reviewer response for version 3

**DOI:** 10.5256/f1000research.173111.r330859

**Published:** 2024-10-15

**Authors:** Juni Handajani

**Affiliations:** 1 Department of Oral Biology, Faculty of Dentistry, Universitas Gadjah Mada, Yogyakarta, Indonesia

I really appreciate your revision of the manuscript.

Is the work clearly and accurately presented and does it cite the current literature?

Yes

If applicable, is the statistical analysis and its interpretation appropriate?

No

Are all the source data underlying the results available to ensure full reproducibility?

Yes

Is the study design appropriate and is the work technically sound?

Yes

Are the conclusions drawn adequately supported by the results?

Yes

Are sufficient details of methods and analysis provided to allow replication by others?

Yes

Reviewer Expertise:

Oral Biology, Oral Immunology

I confirm that I have read this submission and believe that I have an appropriate level of expertise to confirm that it is of an acceptable scientific standard.

# Reviewer report: Reviewer response for version 1

**DOI:** 10.5256/f1000research.169763.r312870

**Published:** 2024-09-24

**Authors:** Heni Susilowati

**Affiliations:** 1 Department if Oral Biology, Faculty of Dentistry, Universitas Gadjah Mada, Universitas Gadjah Mada, KABUPATEN SLEMAN, DI YOGYAKARTA, Indonesia

Thank you for the opportunity to review this article. This article, like similar articles in other fields of science, has become very rapidly developing, especially after the covid-19 pandemic era. This article is very interesting and quite easy to understand, analyzing aspects related to research on dental caries.

 This article is an analytical review of caries research using machine learning that focuses on a specific area, Southeast Asia. 

 Given that caries is a dental and oral disease with a very high prevalence in this region, efforts to prevent it are highly needed, 

 one of which is by developing and improving the quality of caries research. 

 Thus, this article will make an important contribution to the sustainability of caries prevention efforts and innovation of caries prevention methods in this region.

**Major points:**

 The previous article, written by Reyes et al 2022 [Ref 1], has been published in the journal Caries Research 2022; 56(3):161-170. In this regard, we suggest that the author can explain the differences in the principles of their writing with that article.  

**Minor points:**

 This article states that in Southeast Asia, research on dental caries using machine learning techniques is still limited; however, this is not clear compared to countries outside Southeast Asia. If the author adds this analysis to the Discussion section, it will certainly increase the contribution of the article.

 I do hope that this review may add positive improvement to the article.

Is the work clearly and accurately presented and does it cite the current literature?

Yes

If applicable, is the statistical analysis and its interpretation appropriate?

Not applicable

Are all the source data underlying the results available to ensure full reproducibility?

Yes

Is the study design appropriate and is the work technically sound?

Yes

Are the conclusions drawn adequately supported by the results?

Yes

Are sufficient details of methods and analysis provided to allow replication by others?

Yes

Reviewer Expertise:

Oral Microbiology and Immunology

I confirm that I have read this submission and believe that I have an appropriate level of expertise to confirm that it is of an acceptable scientific standard, however I have significant reservations, as outlined above.

## References

[1] : Machine Learning in the Diagnosis and Prognostic Prediction of Dental Caries: A Systematic Review. *Caries Research* .2022;56(3) : 10.1159/000524167 161-170 10.1159/000524167 35636386

# Response

**Published:** 2024-10-07

**Authors:** Hendrik Setia Budi

**Affiliations:** Airlangga University, Indonesia

Response to the reviewer 2 comment:

 Thank you for the opportunity to review this article. This article, like similar articles in other fields of science, has become very rapidly developing, especially after the covid-19 pandemic era. This article is very interesting and quite easy to understand, analyzing aspects related to research on dental caries. This article is an analytical review of caries research using machine learning that focuses on a specific area, Southeast Asia. Given that caries is a dental and oral disease with a very high prevalence in this region, efforts to prevent it are highly needed, one of which is by developing and improving the quality of caries research. Thus, this article will make an important contribution to the sustainability of caries prevention efforts and innovation of caries prevention methods in this region.

**Response:** Thank you for your thorough review. We carefully considered your suggestions and comments and revised our manuscript accordingly. The following are our specific responses to each of the comments. All changes were highlighted in red. We hope the revisions are satisfactory and that our responses adequately address the comments.

 Major points:

 The previous article, written by Reyes et al 2022 [Ref 1], has been published in the journal Caries Research 2022; 56(3):161-170. In this regard, we suggest that the author can explain the differences in the principles of their writing with that article. 

 References

 1. Reyes L, Knorst J, Ortiz F, Ardenghi T: Machine Learning in the Diagnosis and Prognostic Prediction of Dental Caries: A Systematic Review. Caries Research. 2022; 56 (3): 161-170 Publisher Full Text

**Response:** We have added statements in the Introduction section highlighting the gaps addressed by the current systematic review.

 Minor points:

 This article states that in Southeast Asia, research on dental caries using machine learning techniques is still limited; however, this is not clear compared to countries outside Southeast Asia. If the author adds this analysis to the Discussion section, it will certainly increase the contribution of the article.

 I do hope that this review may add positive improvement to the article.

**Response:** We have included a paragraph in the Discussion section that provides a comparative analysis of the application of machine learning for dental caries research between Southeast Asia and countries outside the region.

# Reviewer report: Reviewer response for version 1

**DOI:** 10.5256/f1000research.169763.r315857

**Published:** 2024-09-13

**Authors:** Juni Handajani

**Affiliations:** 1 Department of Oral Biology, Faculty of Dentistry, Universitas Gadjah Mada, Yogyakarta, Indonesia

This study is interesting to to give insight about a bibliometric analysis however more explanation should be added in detail.

 In the background, an explanation of the importance of bibliometric analysis for researchers and clinicians should be added. 

 In the methods, the data is explained using the latest data from 2002-2024. It is suggested to add the reasons for selecting the data.

 This study has explained the importance of bibliometric analysis in tracking public health issues such as dental caries through the application of machine learning. However, it is necessary to explain in more detail how the results of this analysis can give suggestion or recommendation to solve the health problems related to the oral health issues.

Is the work clearly and accurately presented and does it cite the current literature?

Yes

If applicable, is the statistical analysis and its interpretation appropriate?

No

Are all the source data underlying the results available to ensure full reproducibility?

Yes

Is the study design appropriate and is the work technically sound?

Yes

Are the conclusions drawn adequately supported by the results?

Yes

Are sufficient details of methods and analysis provided to allow replication by others?

Yes

Reviewer Expertise:

Oral Biology, Oral Immunology

I confirm that I have read this submission and believe that I have an appropriate level of expertise to confirm that it is of an acceptable scientific standard, however I have significant reservations, as outlined above.

# Response

**Published:** 2024-09-22

**Authors:** Hendrik Setia Budi

**Affiliations:** Airlangga University, Indonesia

**Response to the reviewer comments:**

 This study is interesting to to give insight about a bibliometrics analysis however more explanation should be added in detail. In the background, an explanation of the importance of bibliometrics analysis for researchers and clinicians should be added.

**Response:** we added the explanation in the last paragraph of introduction.

 In the methods, the data is explained using the latest data from 2002-2024. It is suggested to add the reasons for selecting the data.

**Response:** I appreciate the recommendation. The paper has been revised to elucidate the rationale for selecting data from 2002 to 2024. This timeframe was selected because of technological advancements commencing in 2002, signifying a crucial transformation in research methodologies and data accessibility, enabling the identification of pertinent trends in the domain. Incorporating data through 2024 guarantees the inclusion of the most recent advancements and offers a thorough examination of emerging research. This timeframe offers dependable and accessible data, crucial for a comprehensive bibliometrics investigation. This clarification has been incorporated into the Methods section.

 This study has explained the importance of bibliometrics analysis in tracking public health issues such as dental caries through the application of machine learning. However, it is necessary to explain in more detail how the results of this analysis can give suggestion or recommendation to solve the health problems related to the oral health issues.

**Response:** Thank you for the insightful comment. Consequently, we have broadened our discussion to elucidate how the findings of the bibliometrics analysis can offer recommendations for tackling oral health concerns, including dental caries.

*“The bibliometrics analysis highlights significant research trends, pivotal studies, and deficiencies in the existing literature, which can guide evidence-based public health interventions. By identifying the most commonly cited therapies or successful public health initiatives in dental caries prevention, researchers and policymakers can prioritize these techniques for widespread implementation. The machine learning methodology facilitates the discovery of emerging research domains, enabling the prompt recognition of novel therapies or preventive strategies. This can inform funding distribution and policy formulation, ensuring resources are allocated to the most effective strategies for enhancing oral health outcomes.”*

 This elaborated explanation has been incorporated into the discussion to illustrate how bibliometrics insights might yield effective recommendations for tackling oral health issues.
